# The conjunctival microbiome in health and trachomatous disease: a case control study

**DOI:** 10.1186/s13073-014-0099-x

**Published:** 2014-11-15

**Authors:** Yanjiao Zhou, Martin J Holland, Pateh Makalo, Hassan Joof, Chrissy h Roberts, David CW Mabey, Robin L Bailey, Matthew J Burton, George M Weinstock, Sarah E Burr

**Affiliations:** The Genome Institute, Washington University, St Louis, MO 63108 USA; Department of Pediatrics, Washington University School of Medicine, St Louis, MO 63130 USA; Department of Clinical Research, London School of Hygiene and Tropical Medicine, London, WC1E 7HT UK; Disease Control and Elimination Theme, Medical Research Council Unit, Fajara, POB273 The Gambia; The Jackson Laboratory for Genomic Medicine, Farmington, CT 06030 USA

## Abstract

**Background:**

Trachoma, caused by *Chlamydia trachomatis*, remains the world’s leading infectious cause of blindness. Repeated ocular infection during childhood leads to scarring of the conjunctiva, in-turning of the eyelashes (trichiasis) and corneal opacity in later life. There is a growing body of evidence to suggest non-chlamydial bacteria are associated with clinical signs of trachoma, independent of *C. trachomatis* infection.

**Methods:**

We used deep sequencing of the V1-V3 region of the bacterial 16S rRNA gene to characterize the microbiome of the conjunctiva of 220 residents of The Gambia, 105 with healthy conjunctivae and 115 with clinical signs of trachoma in the absence of detectable *C. trachomatis* infection. Deep sequencing was carried out using the Roche-454 platform. Sequence data were processed and analyzed through a pipeline developed by the Human Microbiome Project.

**Results:**

The microbiome of healthy participants was influenced by age and season of sample collection with increased richness and diversity seen in younger participants and in samples collected during the dry season. Decreased diversity and an increased abundance of *Corynebacterium* and *Streptococcus* were seen in participants with conjunctival scarring compared to normal controls. Abundance of *Corynebacterium* was higher still in adults with scarring and trichiasis compared to adults with scarring only.

**Conclusions:**

Our results indicate that changes in the conjunctival microbiome occur in trachomatous disease; whether these are a cause or a consequence is yet unknown.

**Electronic supplementary material:**

The online version of this article (doi:10.1186/s13073-014-0099-x) contains supplementary material, which is available to authorized users.

## Background

Trachoma, caused by the bacterium *Chlamydia trachomatis*, is characterized by recurrent episodes of chronic follicular conjunctivitis. Repeated infection during childhood can lead to scarring of the conjunctiva and the blinding complications of trachomatous trichiasis (TT) and corneal opacification in later life. Persistent, severe inflammation is a contributing factor to progressive scarring yet ocular *C. trachomatis* infection is rarely detected in individuals with scarring.

There is increasing evidence to suggest non-chlamydial pathogens are associated with trachomatous disease. A cross-sectional survey of trachomatous inflammation-follicular (TF) in a low endemicity setting in Tanzania found that children with clinical signs of the disease were more likely to have non-chlamydial bacteria in their eyes than were children without disease; *Streptococcus pneumoniae* and *Haemophilus influenzae* were strongly associated with TF [1]. This finding was independently validated in The Gambia, where *S. pneumoniae* and *H. influenzae* were associated with TF following a mass drug administration campaign for trachoma control [2]. Case-control studies in The Gambia and Tanzania have also shown that the presence of bacterial pathogens in the eye is associated with trachomatous scarring (TS) and TT, an association strengthened with increasing disease severity, as measured by the number of eyelashes touching the eye [3-5]. Non-chlamydial bacteria in the eye have also been shown to be independently associated with TT in Ethiopia [6]. It has been suggested, therefore, that non-chlamydial bacterial infection contributes to the maintenance of an inflammatory state thereby driving the scarring process [5]. This is supported by data from longitudinal studies in The Gambia, which have provided some evidence that non-chlamydial bacterial infection, host inflammatory gene expression and clinical inflammation are associated with recurrence of TT following surgery [3].

While the above studies give some insight into the association of non-chlamydial pathogens with trachomatous disease, they are all limited by the identification of pathogens by bacterial culture, which relies on the ability to grow bacteria under routine laboratory conditions. Deep sequencing of the bacterial gene that encodes the 16S ribosomal RNA subunit (*rrs* or 16S rRNA gene) enables the study of entire bacterial communities using DNA isolated directly from clinical samples [7], thereby offering a more complete picture of the bacterial ecology of the conjunctiva. Studies utilizing 16S rRNA gene sequencing to characterize pathologies at other body sites have shown that alterations in the composition of the microbiome are associated with disease [8,9]. This study aimed to characterize the microbiome of the conjunctiva of individuals living in a trachoma-endemic community and to identify changes in the bacterial community structure, richness and diversity associated with trachomatous disease.

## Methods

### Ethical permission

This study adhered to the tenets of the Declaration of Helsinki. Approval was obtained from the Gambian Government/Medical Research Council Unit, The Gambia Joint Ethics Committee. Written, informed consent was obtained from all participants at the time of sample collection. In the case of children, consent was obtained from a parent or guardian.

### Study participants

Samples were retrospectively drawn from an archive built up from individuals recruited in communities across The Gambia, West Africa. Cases of active or scarring trachoma were identified from screening records, community ophthalmic nurse referral and opportunistic rapid screening. Control individuals with normal conjunctivae were selected by matching for age, sex, ethnicity and location.

### Trachoma grading

Participating individuals were examined for clinical signs of trachoma in the field and high resolution digital photographs were taken of each conjunctival surface at the time of sample collection. An FPC score was then assigned to each sample by an ophthalmologist who graded the photographs according to the 1981 WHO Trachoma Grading System (FPC, for follicles, papillae, cicatricae) [10]. Any sample for which there was no photograph or for which the photograph could not be accurately graded was excluded. For analyses, the presence of follicles was defined as an F score >0. Conjunctival scarring was defined as a C score >0. Participants with normal, healthy conjunctivae, as defined by a score of F0P0C0, served as controls.

### Sample collection and processing

Samples were collected between February 2009 and April 2011. Samples were taken from the upper tarsal conjunctiva using Dacron swabs and stored in 250 μl RNA*later* (Ambion, Life Technologies, Carlsbad, CA, USA) on ice blocks in the field. Upon return to the laboratory, samples were archived at -20°C until processing. Total, genomic DNA was extracted using the PowerSoil DNA Isolation Kit (Mo Bio Laboratories, Carlsbad, CA, USA) according to the manufacturer’s instruction. The presence of *C. trachomatis* DNA was assayed using the Amplicor CT/NG assay (Roche Molecular Systems, Branchburg, NJ, USA) according to modifications previously described [11].

The V1-V3 region of the 16S rRNA gene was amplified using primers 27 F (5′-AGAGTTTGATCCTGGCTCAG-3′) and 534R (5′- ATTACCGCGGCTGCTGG-3′). Primers also contained an adaptor sequence and one of 96 tags unique to each sample. PCR was performed with the following conditions: 30 cycles of 95°C 2 minutes; 56°C 0.5 minutes and 72°C 5 minutes. Amplicons were purified, pooled at equimolar concentrations and sequenced by pyrosequencing on the Roche-454 titanium platform using the protocol developed by the Human Microbiome Project [12]. Sequence data were submitted to the Sequence Read Archive (SRA) at the National Center for Biotechnology Information (NCBI) under accession number PRJNA248889.

Reagent and non-template controls were extracted and sequenced according to the same procedure and generated 63 to 236 reads. The major taxon present in these controls was *Ralstonia*.

### Sequence data processing

Data processing and quality control (QC) were performed according to standardized protocols developed by the Human Microbiome Project [12]. Briefly, samples were demuxed allowing one mismatch in the barcodes. Reads were filtered to remove those samples with average quality scores <35 and/or read length less than 200 nucleotides. Chimeric sequences were removed using Chimera-Slayer [13]. Following initial QC, samples with a read depth of less than 1,000 were re-sequenced. Reads passing QC were then classified from phylum to genus level using the Ribosomal Database Project Naive Bayesian Classifier (version 2.2, training set 6) [14]. Taxa assigned with <0.5 confidence threshold were reassigned to the next higher taxonomic level in which the classification threshold was >0.5.

### Analysis

After data processing, a taxonomical matrix was constructed (rows as genera and columns as samples) and then rarefied to 1,000 reads using the Vegan package in R [15]. Multidimensional scaling (MDS) with the Bray-Curtis index was used to explore bacterial community structure. Data were visualized using the MASS package in R [16]. Permutational multivariate analysis of variance (PERMANOVA) [17] was used to test whether bacterial community structure differed between variables using the Vegan package in R [15]. Metastats was used to identify genera that contributed to the difference between two bacterial communities [18]; genera were considered significantly different if the q value ≤0.1 and if the mean relative abundance for a given genus was at least 1% in one group. Wilcoxon rank sum test was used to test the differences in richness and Shannon diversity between two groups.

## Results

### Characteristics of samples and sequencing reads

Upper tarsal conjunctival swabs collected from 260 participants (130 case-control pairs), were processed for 16S rRNA gene sequence analysis. Following post-sequencing QC measures, 220 samples (84%) were retained for analysis. Of these, 105 samples were from individuals with normal healthy conjunctiva (F0P0C0); the remaining 115 participants had clinical signs of trachoma. Three children with normal conjunctiva, but no children with signs of trachoma and no adults, had evidence of ocular *C. trachomatis* infection by Amplicor CT/NG PCR. Demographic characteristics for the 220 samples included in the final data set are given in Table 1.

**Table 1:** **Demographic characteristics of study participants with and without trachomatous disease**

**Variable**		**Clinical signs**		
		**Cases**	**Controls**	**Total**
**Age**	≤10 years	29	21	50
	>10 years	86	84	170
**Sex**	Female	75	72	147
	Male	40	33	73
**Season**	Dry	63	63	126
	Wet	52	42	94
**Region**	Banjul	1	1	2
	Western Division	57	56	113
	Lower River Division	35	27	62
	Central River Division	6	6	12
	Upper River Division	8	7	15
	North Bank Division	8	8	16
**Ethnicity**	Wolof	15	13	28
	Mandinka	41	38	79
	Jola	36	35	71
	Fula	11	7	18
	Serere	1	0	1
	Manjago	3	2	5
	Balanta	1	0	1
	Bambara	0	1	1
	Other/Unknown	7	9	16

The resulting dataset generated 1,690,427 reads with an average read depth per sample of 7,684 ± 4,909. In total, 24 phyla, 41 classes, 94 orders, 188 families and 880 genera were identified. At the genus level, 14.2% of reads were unclassified.

### Taxon abundance

**Figure 1:**
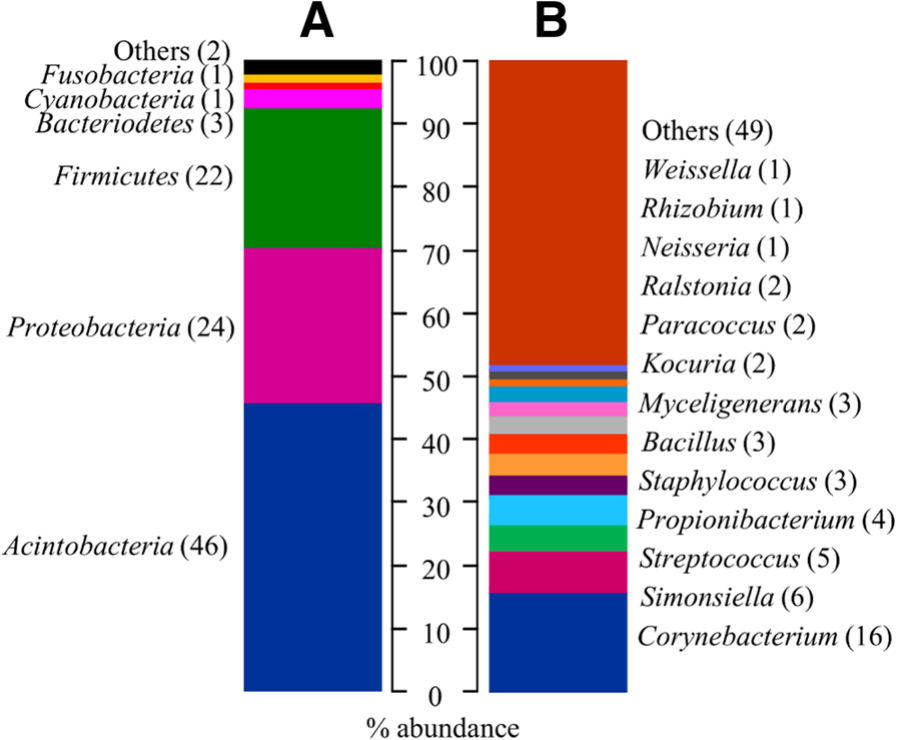
**Relative abundance of major taxa found in the normal healthy conjunctiva. (A)** Major phyla; **(B)** major genera. The abundance of each taxa is represented as a percentage of the total number of reads obtained from participants with normal conjunctivae (F0P0C0). Less abundant taxa (<1%) are grouped together as ‘Others’.

Analysis of sequence data from the 105 participants with normal healthy conjunctivae (F0P0C0) revealed a highly diversified bacterial community. After rarefying all samples to 1,000 reads, 610 genera belonging to 22 phyla were identified. Three dominant phyla, Actinobacteria, Proteobacteria and Firmicutes, accounted for 46%, 24% and 22% of the total bacterial community, respectively (Figure 1A). At the genus level, 13 genera were present at more than 1% relative abundance (Figure 1B). Of these, six were shared by at least 80% of all samples and together accounted for more than a third of the entire bacterial community characterized: *Corynebacterium*, *Streptococcus*, *Propionibacterium*, *Bacillus*, *Staphylococcus* and *Ralsontia. Corynebacterium* was the most abundant genus, representing 16.2% of all reads and was found in all samples from healthy conjunctivae.

### Factors influencing the conjunctival microbiome

**Figure 2:**
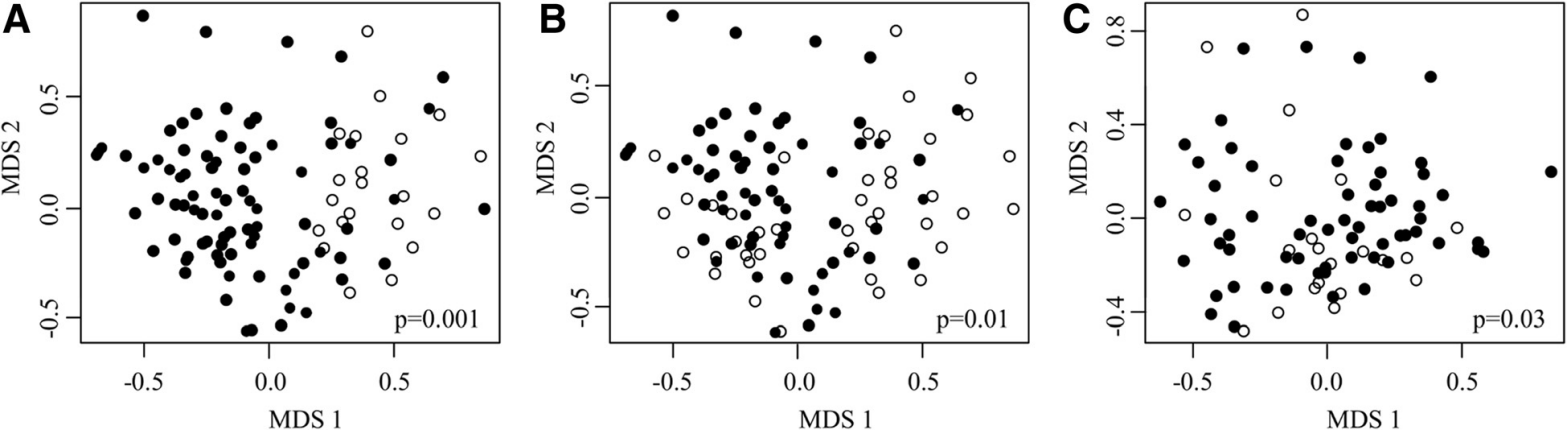
**Factors influencing bacterial community structure of normal conjunctivae as represented by multidimensional scaling. (A)** Stratification of all participants with normal conjunctivae (F0P0C0) by age with those ≤10 years represented by open circles and those >10 years by filled circles. **(B)** Stratification of all participants with normal conjunctivae (F0P0C0) by season: open circles represent samples collected during the wet season, filled circles represent samples collected during the dry season. **(C)** Stratification of only participants aged >10 years with normal conjunctivae (F0P0C0) by season of sample collection: open circles represent samples collected during the wet season, filled circles represent samples collected during the dry season. *P*-values generated by PERMANOVA.

In an effort to determine factors influencing the conjunctival microbiome in our sample set, we performed multivariable analysis using PERMANOVA. The model included age (≤10 or >10 years), sex, season of sample collection (dry or wet), geographical location (by region) and ethnicity. Bacterial community structure (composition and abundance in one sample or a group of samples) was compared between groups while controlling for the other variables. As illustrated in Figure 2A, stratification of subjects with normal conjunctivae (F0P0C0) by age (≤10 or >10 years) resulted in the formation of two distinct groups; bacterial community structure between the two was significantly different (*P* = 0.001). Seasonality was also found to exert a strong influence as shown in Figure 2B; the bacterial community structure of normal conjunctivae sampled in the dry versus wet seasons are significantly different (*P* = 0.01). This effect was still apparent when comparing bacterial community structure of only participants aged >10 years sampled in the dry versus wet seasons (*P* = 0.03) (Figure 2C). In contrast, geographical location, gender and ethnicity did not have a significant effect (geographical regions, *P* = 0.18; gender, *P* = 0.29; ethnicity, *P* = 0.80) (Additional files 1, 2 and 3).

We compared richness (absolute number of taxa present) and Shannon diversity indices (number and relative abundance of each taxa) as measures of the complexity of the bacterial communities in the younger and older age groups. Both richness (*P* = 0.03) and Shannon diversity (*P* = 0.03) were significantly higher in the children aged ≤10 years with normal conjunctivae (F0P0C0) than in the older participants (Additional file 4) with children harboring, on average, 20 more genera than older individuals.

Metastats was used to identify genera present at differing abundance between the younger and older age groups with healthy conjunctivae (F0P0C0). *Corynebacterium*, *Propionibacterium*, *Myceligenerans*, *Paracoccus* and two unclassified genera from the Promicromonosporaceae family and Actinomycetales order were more abundant in the older group (Table 2), with 13.4 times more Actinomycetales being found in these participants compared to children aged ≤10 years. The abundances of five genera (*Streptococcus*, *Kocuria*, *Staphylococcus*, *Micrococcus* and *Brachybacterium*) were significantly higher in the ≤10 year age group (Table 2) with the abundance of *Streptococcus* in children 6.2 times higher than in older participants.

**Table 2:** **Changes in taxa abundance between groups**

**Taxanomic classification (phylum, class, order, family, genus)**	**Relative abundance (median (range))**		**q-value**
	**Group 1**	**Group2**	
**Group 1 ≤ 10 years; group 2 > 10 years (normal conjunctivae; F0P0C0)**			
*Firmicutes; Bacilli; Lactobacillales; Streptococcaceae; Streptococcus*	8.0 (0.1-42.6)	1.3 (0-73.3)	0.012
*Actinobacteria; Actinobacteria; Actinomycetales; Micrococcaceae; Kocuria*	4.6 (0-23.9)	0.3 (0-23.4)	0.007
*Firmicutes; Bacilli; Bacillales; Staphylococcaceae; Staphylococcus*	5.7 (1.0-20.3)	2.0 (0-21.4)	0.010
*Actinobacteria; Actinobacteria; Actinomycetales; Micrococcaceae; Micrococcus*	3 (0-6.3)	0 (0-6.0)	0.003
*Actinobacteria; Actinobacteria; Actinomycetales; Dermabacteraceae; Brachybacterium*	0.9 (0-6.4)	0.2 (0-4.0)	0.027
*Actinobacteria; Actinobacteria; Actinomycetales; Corynebacteriaceae; Corynebacterium*	9.1 (1.0-29.3)	13.5 (0.4-67.6)	0.016
*Actinobacteria; Actinobacteria; Actinomycetales; Promicromonosporaceae;* unclassified	0	0.8 (0-5.8)	0.001
*Proteobacteria; Alphaproteobacteria; Rhodobacterales; Rhodobacteraceae; Paracoccus*	0.7 (0-1.6)	1.6 (0-10.6)	0.001
*Actinobacteria; Actinobacteria; Actinomycetales; Propionibacteriaceae; Propionibacterium*	0.5 (0-3.6)	1.7 (0-40.3)	0.001
*Actinobacteria; Actinobacteria; Actinomycetales; Promicromonosporaceae; Myceligenerans*	0 (0-0.1)	3.0 (0-17.5)	0.001
*Actinobacteria; Actinobacteria; Actinomycetales;* unclassified	0.8 (0-2.5)	10.7 (0-40.2)	0.001
**Group 1, dry season; group 2, wet season (normal conjunctivae; F0P0C0)**			
*Firmicutes; Bacilli; Bacillales; Bacillaceae; Bacillus*	3.7 (0-16.3)	0.3 (0-2.8)	0.005
*Firmicutes; Bacilli; Bacillales; Bacillaceae; Tumebacillus*	0.6 (0-11.4)	0 (0-1.7)	0.007
**Group 1, F > 0; group 2 F0P0C0 (wet season)**			
No significant differences			
**Group 1, all C > 0; group 2, C = 0 (dry season)**			
*Actinobacteria; Actinobacteria; Actinomycetales; Corynebacteriaceae; Corynebacterium*	18.0 (0-88.0)	11.1 (0.4-60.1)	0.034
*Firmicutes; Bacilli; Lactobacillales; Aerococcaceae; Globicatella*	0 (0-3.9)	0.5 (0-6.2)	0.043
*Firmicutes; Bacilli; Lactobacillales; Streptococcaceae; Streptococcus*	2.2 (0-90.6)	1.4 (0-24.5)	0.040
**Group 1, all C > 0; group 2, C = 0 (wet season)**			
No significant differences			
**Group 1, C > 0; group 2, C > 0 + TT (dry season)**			
*Actinobacteria; Actinobacteria; Actinomycetales; Corynebacteriaceae; Corynebacterium*	12.2 (0.1-78.4)	26.8 (0-88.0)	0.043
*Actinobacteria; Actinobacteria; Actinomycetales; Micrococcaceae; Kocuria*	0 (0-6.8)	0.2 (2-2.2)	0.047
*Actinobacteria; Actinobacteria; Actinomycetales; Promicromonosporaceae; Myceligenerans*	2.2 (0-10.5)	0 (0-12.1)	0.048
*Actinobacteria; Actinobacteria; Actinomycetales; Promicromonosporaceae;* unclassified	0.8 (0-3.2)	0 (0-4.6)	0.047
*Actinobacteria; Actinobacteria; Actinomycetales;* unclassified	6.9 (0-32.3)	1.2 (0-31.5)	0.006
*Proteobacteria; Alphaproteobacteria; Rhodobacterales; Rhodobacteraceae; Paracoccus*	1.4 (0-8.4)	0.1 (0-7.5)	0.026
**Group 1, C > 0; group 2, C > 0 + TT (wet season)**			
No significant differences			

We characterized differences in the microbiome associated with seasonal change following stratification by age. Richness (*P* = 0.006) and Shannon diversity (*P* = 0.004) were significantly higher in older participants (aged >10 years) sampled during the dry season (Additional file 5). The genera *Bacillus* and *Tumebacillus* were more abundant in the dry season (Table 2). All samples from the younger age group (≤10 years) were collected during the wet season therefore, no seasonal effect could be analyzed in this age group.

### Changes in the conjunctival microbiome associated with trachoma

**Figure 3:**
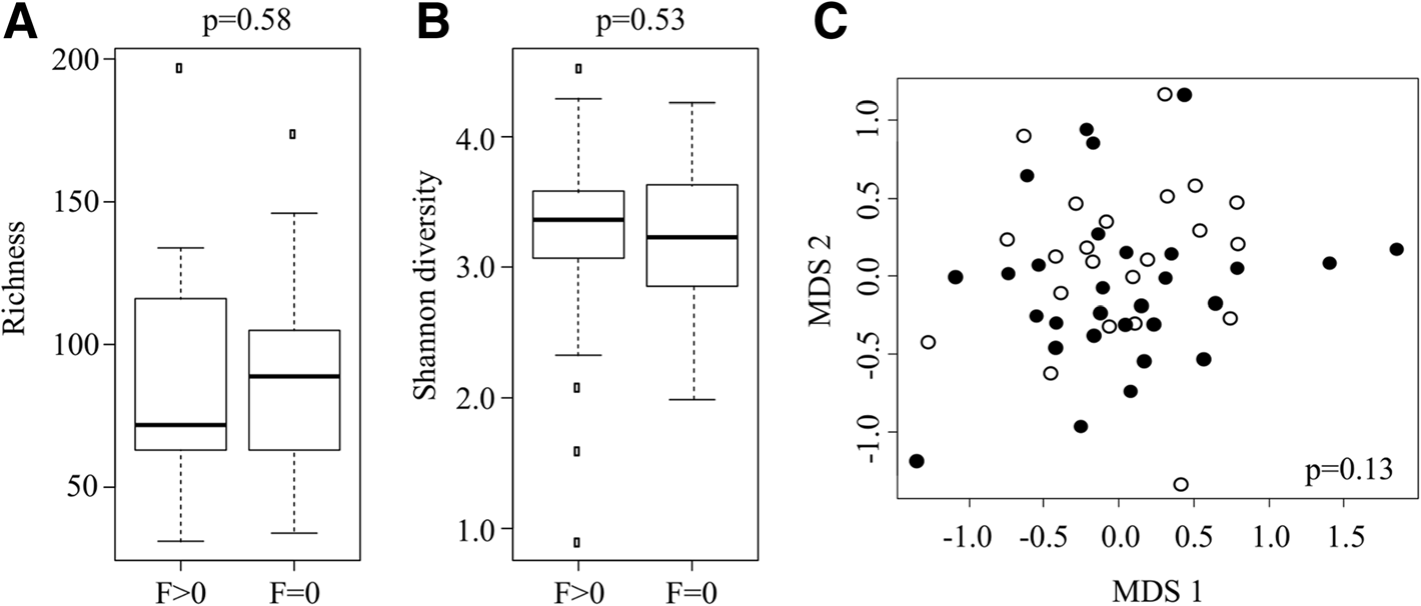
**Differences in richness, diversity and community structure associated with follicles in children. (A,B)** Boxplots indicate the distribution of richness **(A)** and Shannon diversity measures **(B)** in children with follicles (F > 0) compared with those with normal conjunctivae (F0P0C0); *P*-values calculated using Wilcoxon rank sum test. **(C)** Differences in bacterial community structure between children with follicles (F > 0, filled circles) and children with normal conjunctivae (F0P0C0, open circles) visualized by MDS; *P*-value was generated by PERMANOVA.

We first compared changes in the community structure in children with normal conjunctivae (F0P0C0) versus those with signs of follicles as defined by an F score >0. One child with an FPC score of F0P3C0 was also included as a case in this analysis. Richness and Shannon diversity measures did not vary significantly between groups (richness, *P* = 0.58; diversity, *P* = 0.53; Figure 3A, B) nor was bacterial community structure significantly different as shown by MDS (Figure 3C) and PERMANOVA analysis *P* = 0.13). *Haemophilus* was present in higher abundance in children with trachoma than in normal controls (*P* = 0.023); however, this finding was not significant when corrected for multiple comparisons (q = 0.291). Indeed, the increased abundance in cases was largely driven by one child with intense inflammation (F2P3C0) and a relative abundance of *Haemophilus* of 60%.

**Figure 4:**
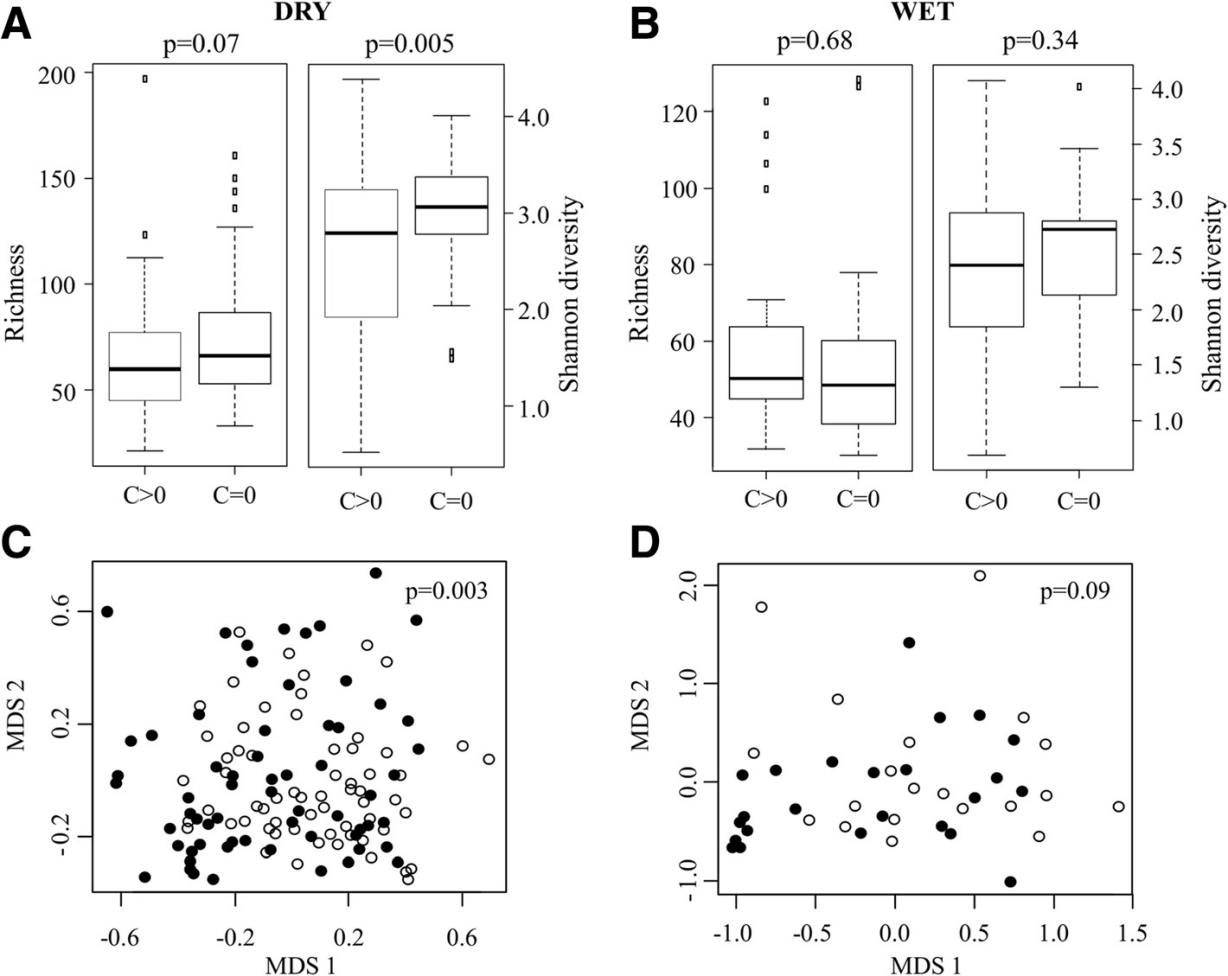
**Differences in richness, diversity and community structure associated with conjunctival scarring. (A,B)** Boxplots indicate distribution of richness and Shannon diversity measures in participants aged >10 years with conjunctival scarring (C > 0) versus those with normal conjunctivae (F0P0C0) sampled in the dry **(A)** and wet **(B)** seasons; *P*-values calculated using Wilcoxon rank sum test. **(C,D)** MDS was used to visualize differences in community structure between all participants aged >10 years with scarring (C > 0, filled circles) versus those with normal conjunctivae (F0P0C0, open circles) sampled during the dry season **(C)** and all participants aged >10 years with conjunctival scarring (C > 0, filled circles) versus those with normal conjunctivae (F0P0C0, open circles) sampled during the wet season **(D)**; *P*-values generated by PERMANOVA.

Community structure was compared between participants aged >10 years with normal conjunctivae (F0P0C0) and those with clinical signs of conjunctival scarring (C > 0 with and without TT) following stratification by season. Higher diveristy was found in participants with normal healthy conjunctivae during the dry season (*P* = 0.005; Figure 4A) but not during the wet season (*P* = 0.34; Figure 4B). MDS and PERMANOVA analysis indicated the community structure was significantly different between all participants with conjunctival scarring and normal controls in the dry (*P* = 0.003; Figure 4C) but not the wet season (*P* = 0.09; Figure 4D). In the dry season, the abundance of *Corynebacterium* and *Streptococcus* were higher in the participants with conjunctival scarring than in normal controls (Table 2). Abundance of *Corynebacterium* was also higher in samples with conjunctival scarring collected during the wet season but this did not reach statistical significance (*P* = 0.037, q = 0.170).

**Figure 5:**
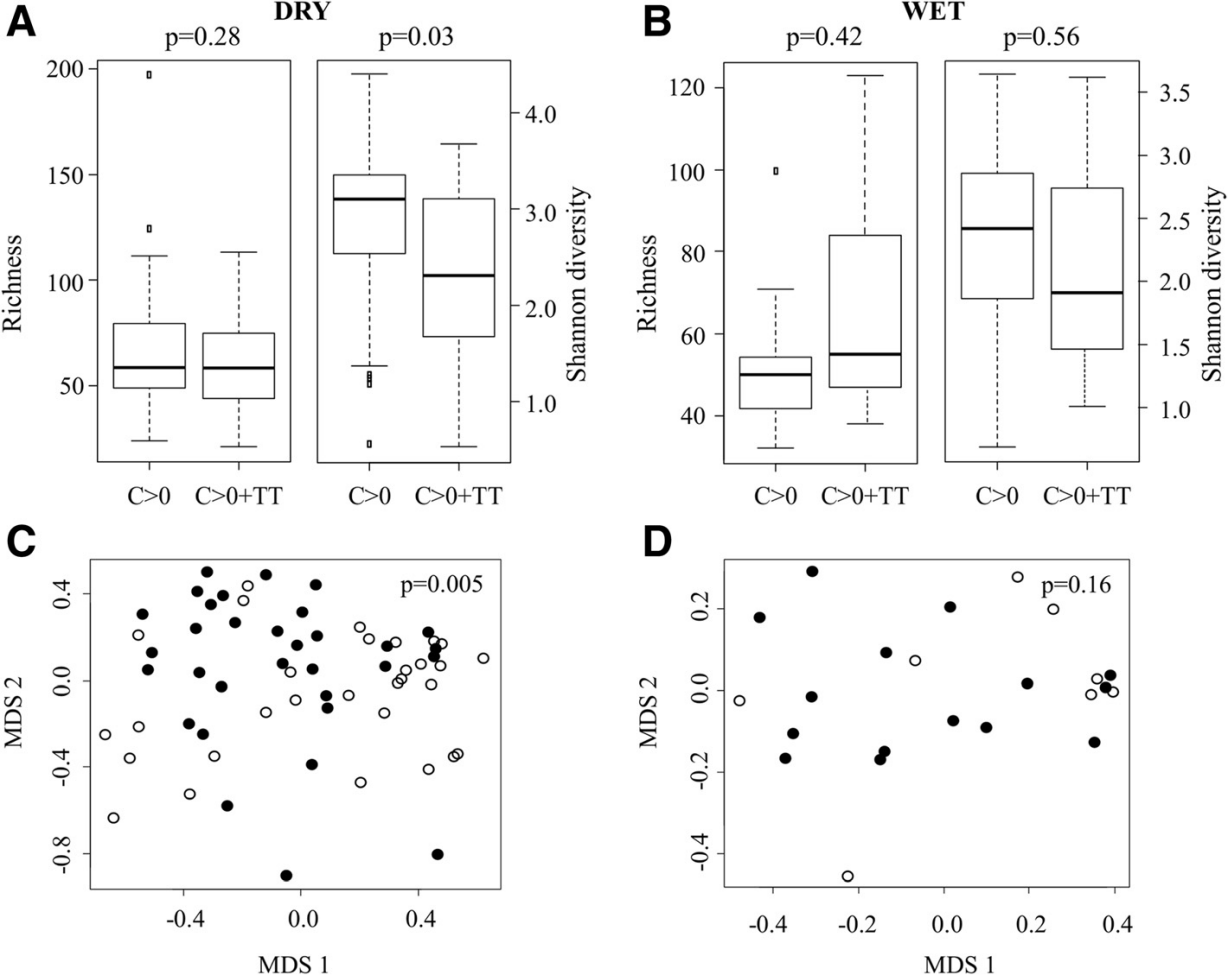
**Differences in richness, diversity and community structure associated with trichiasis. (A,B)** Boxplots indicate distribution of richness and Shannon diversity measures in participants aged >10 years with conjunctival scarring only versus those with scarring and TT sampled in the dry **(A)** and wet **(B)** seasons; *P*-values calculated using Wilcoxon rank sum test. **(C,D)** MDS was used to visualize differences in community structure between participants aged >10 years of age with scarring only (C > 0, filled circles) versus those with scarring and TT (C > 0 + TT, open circles) sampled during the dry season **(C)** and participants aged >10 years with scarring only (C > 0, filled circles) versus those with scarring and TT (C > 0 + TT, open circles) sampled during the wet season **(D)**; *P*-values generated by PERMANOVA.

We compared the bacterial community structure in participants with conjunctival scarring (C > 0) versus those with scarring and TT (C > 0 + TT). Data were further stratified by season. There was no difference in the number of genera detected in conjunctivae with scarring versus scarring and TT in either the dry (*P* = 0.28) or wet (*P* = 0.42) seasons (Figure 5A,B) yet Shannon diversity was significantly higher in conjunctivae with scarring in the dry season (*P* = 0.03; Figure 5A). Bacterial community structure was different between the two groups during the dry season as indicated by MDS plots (Figure 5C) and PERMANOVA analysis (*P* = 0.005) but not during the wet season (Figure 5D; *P* = 0.16). During the dry season, *Corynebacterium* was found at higher abundance in participants with scarring and TT (Table 2).

## Discussion

The data-set described here represents the largest description of the conjunctival microbiome, defined by deep-sequencing of the 16S rRNA gene, to date. Inter-variation of a given genera was high, which is consistent with observations from other body sites [12]. We identified over 600 genera, the vast majority of which were found at <1% relative abundance considering all sequencing reads obtained from normal healthy conjunctivae. Of those genera found at ≥1% abundance, only six were found in at least 80% of participants with normal conjunctivae, *Corynebacterium*, *Streptococcus*, *Propionibacterium*, *Staphylococcus*, *Bacillus* and *Ralstonia*.

As *Ralstonia* was the major taxon found in our negative controls, we cannot confirm it is a constituent of the ocular flora in this population, although it has been reported in healthy and infected eyes in other settings [19,20]. The presence of the remaining five genera found in the majority of our samples is consistent with descriptions of the ocular microbiota determined by bacterial culture techniques, which have recently been reviewed [21]. Staphylococci are most commonly isolated from ocular swabs followed by *Propionobacterium* sp. and diphtheroid bacteria (including *Corynebacterium* sp.). *Streptococcus* and *Bacillus* species are less frequently isolated [21]. Only one other independent study has characterized the ocular microbiome using the 16S deep-sequencing approach and comparison with our data suggests greater variation in the ocular microbiome may exist between populations than is indicated by bacterial culture; a study of four American volunteers has reported, in addition to *Propionobacterium* and *Corynebacterium*, high relative abundance of *Pseudomonas* (18%), *Bradyrhizobium* (12%), and *Acinetobacter* (9%) [19]. These genera that were not a significant component of the microbiome of our sample set each accounting for less than 1% relative abundance. The higher level of diversity seen in our sample set, in comparison with other populations, may suggest many of the bacteria found on the conjunctiva of Gambians are not indigenous to this niche but introduced through interaction with the local environment.

With respect to the similarity of the ocular microbiome to other body sites, comparisons are limited as the majority of data describing the human microbiome have come from Western populations. Nevertheless, the high abundance of *Corynebacterium* and *Propionibacterium* in our samples suggests the conjunctival microbiome more closely resembles that of the skin than any other body site [22,23]. While Actinobacteria was the dominant phylum in our sample set, representatives of the phyla Proteobacteria and Firmicutes both accounted for approximately one quarter of all reads from healthy conjunctiva. The high abundance of *Streptococcus* and *Staphylococcus* (phylum Firmicutes) in our samples is also characteristic of the skin flora [19,20]. The proteobacteria *Simonsiella* accounted for 6% of the reads from healthy conjunctivae; while high abundance of this genus is a characteristic of the oral cavity and associated sites, these are dominated by representatives of the phylum Firmicutes [22,24,25].

In our sample set, children aged ≤10 years had greater richness and diversity in the bacterial communities of the conjunctiva than older participants. The abundance of *Streptococcus*, in particular, was markedly different between the age groups with significantly higher levels seen in younger participants, which is consistent with the very high prevalence of nasopharyngeal *S. pneumoniae* carriage in Gambian children [26]. A number of factors, including differences in hygiene behaviors, close contact between children and decreased immunity, may explain some of the increased diversity seen in the young age group. However, our ability to draw definitive conclusions with respect to the effect of age on the conjunctival microbiome is limited by the case/control study design as environmental factors associated with trachomatous disease may be over-represented in our control group. While our data suggest differences in the microbiome between children and adults, these should be confirmed in a population-based survey.

The seasonal effect on the microbiome was characterized by higher abundance of the soil-born genera *Bacillus* and *Tumebacillus* in adults during the dry season. These findings suggest that during the dusty conditions typical of The Gambia’s dry season, increased numbers of bacteria are introduced onto the ocular surface through environmental exposure. No children were sampled during the dry season, preventing us from examining the seasonal effect on the younger age group.

As the prevalence of active trachoma has fallen in The Gambia over recent years, so too has the severity of clinical signs with fewer children with large numbers of follicles being seen [27]. In the current study, half of the children diagnosed in the field as having trachoma were judged to have an F score of 1 with little or no inflammation (*P* < 3) when the photographs of their eyelids were viewed by an ophthalmologist. We therefore chose to analyze potential changes in the microbiome of children with follicles (F score >0) versus those with normal eyes. When comparing these groups, no genus was found at increased abundance in the cases when data were corrected for multiple testing. One child with unusually high abundance of *Haemophilus* had signs of severe inflammation, which is consistent with the hypothesis that inflammation caused by non-chlamydial bacterial infection exacerbates clinical signs of disease. Alternatively, this may indicate that inflammation of the conjunctiva as a result of trachoma makes the eye more susceptible to secondary bacterial infection. However, our relatively small group sizes preclude us from examining changes in the microbiome as a function of increasing severity of inflammation.

*Streptococcus pneumoniae* has been found more often in conjunctivae with TF than in normal controls [1,2] yet we did not identify this genus as being significantly different between children with an F score >0 and normal controls. The 16S rRNA gene sequencing method that we have used however, prevents resolution to the species level. Even if increased numbers of *S. pneumoniae* are present in cases compared with controls, a high abundance of non-pneumococcal *Streptococcus* in both groups may mask this association. This explanation is supported by a study in Tanzania that found prevalence of viridans streptococci in ocular samples of children was three-fold higher than that of pneumococci [1].

It has been suggested that in-turned or mis-directed eyelashes may provide a conduit for increased introduction of bacteria into the eye [1,6]. Our results, however, do not support this hypothesis as the number of genera detected in individuals with conjunctival scarring versus scarring and TT was not significantly different. Scarring and TT was associated with a decrease in diversity in the dry season, largely driven by an increase in the abundance of *Corynebacterium* in those with TT versus those with scarring alone. This is not the first time *Corynebacterium* has been documented in trachomatous eyes. A study in Ethiopia comparing the bacterial flora of conjunctivae with TS with those with TT reported a higher prevalence of carriage of *Corynebacterium* in TT [6] while a study in Tanzania found a higher prevalence of carriage in TS compared with normal controls [5]. In both of these studies however, *Corynebacterium* was considered a commensal organism. Clearly the genus *Corynebacterium* is a significant component of the normal flora of the eye in many populations. However, the presence of ‘normal’ flora may not be indicative of a healthy state. The most common example of this is bacterial vaginosis, where imbalance in the normal flora leads to changes in pH and the overgrowth of particular constituents of the normal flora [28]. While it is possible that a similar dysbiosis of the ocular flora is involved in conjunctival scarring, further study will be needed to determine whether this is a cause or an effect of the disease. This might include longitudinal follow-up of participants and characterization of host immune responses known to be associated with the scarring process.

During the dry season, *Streptococcus* was found at higher abundance in adults with scarring than in controls, but not during the wet season, possibly reflecting the lower number of individuals sampled (43 in the wet season versus 126 in the dry). The abundance of *Streptococcus* was not significantly different between only scarring versus scarring and TT cases, despite evidence that suggests *S. pneumoniae* in the eye may be associated with increasing clinical severity, as measured by the number of eyelashes touching the eye [4]. However, this association may be obscured by a high abundance of non-pneumococcal *Streptococcus*.

The potential for contamination of the samples from environmental sources is a limitation of this study. The presence of many soil-borne bacteria, in particular, may be a reflection of environmental contamination during the sampling process. However, the ocular surface is continually exposed to the external environment and while these organisms may not actively colonize the conjunctival surface, it is reasonable to expect they are continually introduced into the eye, particularly in a resource-poor setting such as The Gambia where housing standards, access to sanitation, use of water and public health awareness are low. This is supported by a recent study characterizing the bacterial communities present on the hands of Tanzanian women, which found the bacterial communities were dominated by soil-borne bacteria, including members of the Rhodobacteraceae, Nocardioidaceae, Bacillaceae, Bradyrhizobiaceae and Rhizobiaceae families [29]. In order to minimize the impact of potential environmental contaminants on the measured diversity of our samples, we rarefied all samples to 1,000 reads. We also removed all taxa found at less than 1% relative abundance after rarefaction to further minimize the effect of potential contamination on the community comparisons between groups. Future longitudinal study and bacterial community transcriptomics may help distinguish bacteria that actively colonize, or replicate, on the conjunctival surface from those that are transiently introduced.

## Conclusions

Changes in the bacterial community structure and reduced diversity are associated with trachomatous disease. Further work is needed to determine whether these changes contribute to the scarring process.

## Additional files

Additional file 1:
**Influence of geographical location on bacteria community structure as represented by multidimensional scaling (MDS).** Bacterial community structure visualized by MDS. Participants with normal conjuctivae (F0P0C0) stratified by geographical region as indicated. *P*-values generated by PERMANOVA. Additional file 2:
**Influence of gender on bacteria community structure as represented by multidimensional scaling (MDS).** Bacterial community structure visualized by MDS. Participants with normal conjuctivae (F0P0C0) stratified by gender as indicated. *P*-values generated by PERMANOVA. Additional file 3:
**Influence of ethnicity on bacteria community structure as represented by multidimensional scaling (MDS).** Bacterial community structure visualized by MDS. Participants with normal conjuctivae stratified by ethnicity (F0P0C0) as indicated. *P*-values generated by PERMANOVA. Additional file 4:
**Effect of age on bacterial community richness and diversity.** Boxplots indicate the distribution of **(A)** richness and **(B)** Shannon diversity measures in younger (≤10 years) and older (>10 years) participants with normal conjunctivae (F0P0C0). *P*-values calculated using Wilcoxon rank sum test. Additional file 5:
**Effect of season on bacterial community richness and diversity.** Boxplots indicate the distribution of **(A)** richness and **(B)** Shannon diversity measures in participants >10 years of age with normal conjunctivae (F0P0C0) sampled during the dry and wet seasons. *P*-values calculated using Wilcoxon rank sum test.

## Abbreviations

FPC: follicles, papillae, cicatricae
MDS: multidimensional scaling
PCR: polymerase chain reaction
QC: quality control
TF: trachomatous inflammation-follicular
TS: trachomatous scarring
TT: trachomatous trichiasis
