# Supplementary figures and images for: The conjunctival microbiome in health and trachomatous disease: a case control study
Source: Genome Med. 2014 Nov 15;6(11):99. doi: 10.1186/s13073-014-0099-x (PMC4256740; doi:10.1186/s13073-014-0099-x)

# Additional File 1

Stratification by geographical region

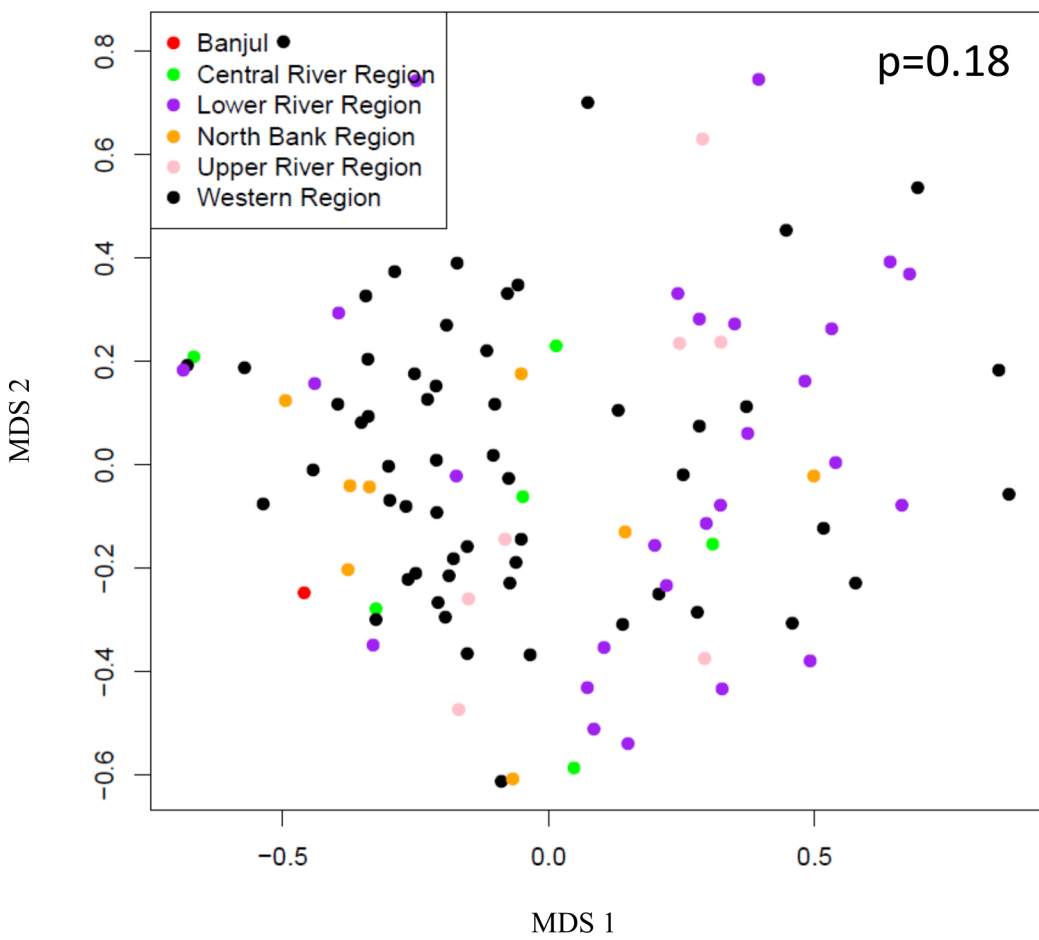

Supplement: Additional file 1: — Influence of geographical location on bacteria community structure as represented by multidimensional scaling (MDS). Bacterial community structure visualized by MDS. Participants with normal conjuctivae (F0P0C0) stratified by geographical region as indicated. P-values generated by PERMANOVA. [file 13073_2014_99_MOESM1_ESM.pdf]

# Additional File 2

Stratification by gender

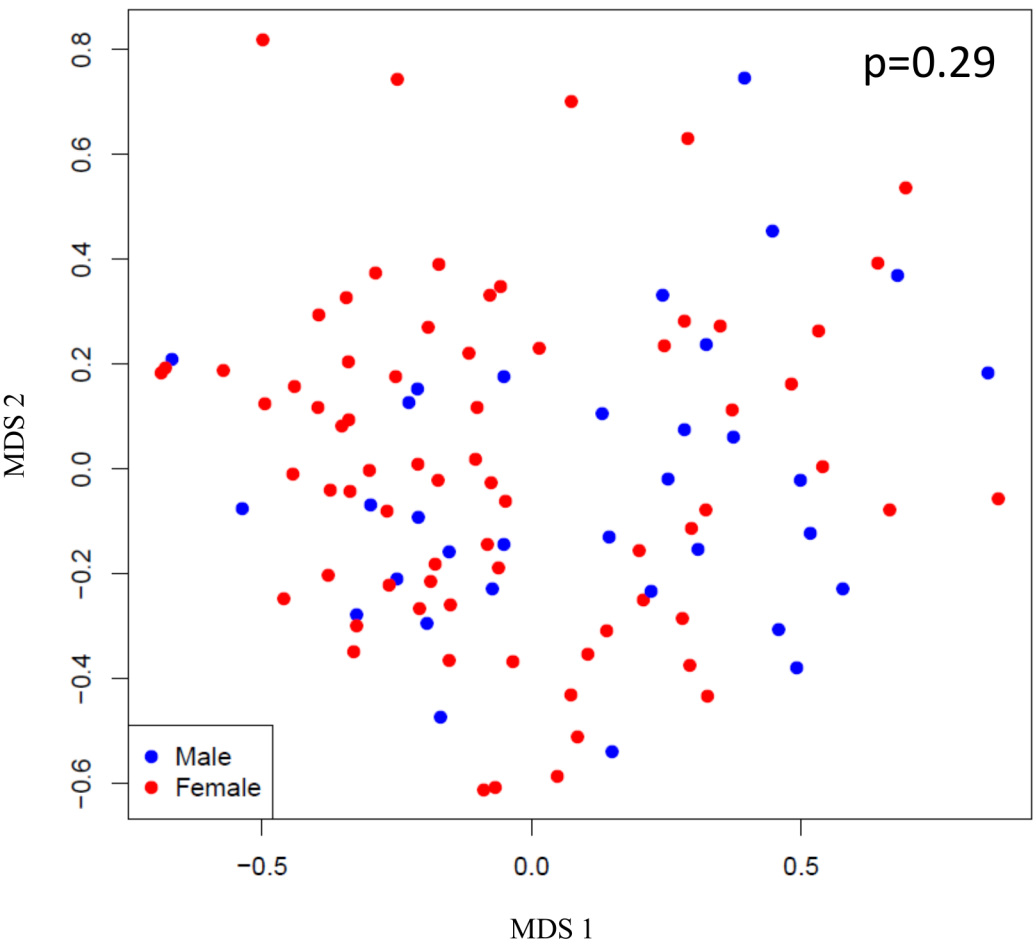

Supplement: Additional file 2: — Influence of gender on bacteria community structure as represented by multidimensional scaling (MDS). Bacterial community structure visualized by MDS. Participants with normal conjuctivae (F0P0C0) stratified by gender as indicated. P-values generated by PERMANOVA. [file 13073_2014_99_MOESM2_ESM.pdf]

# Additional File 3

Stratification by ethnicity

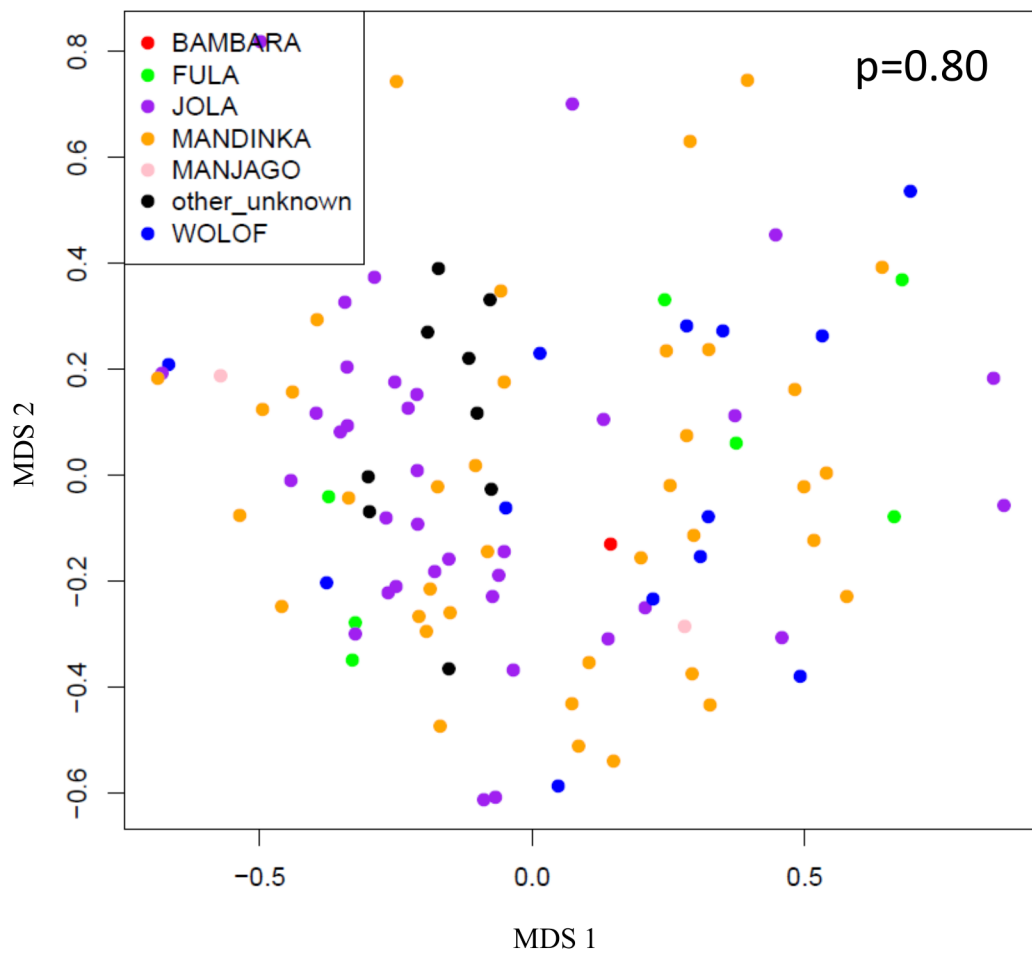

Supplement: Additional file 3: — Influence of ethnicity on bacteria community structure as represented by multidimensional scaling (MDS). Bacterial community structure visualized by MDS. Participants with normal conjuctivae stratified by ethnicity (F0P0C0) as indicated. P-values generated by PERMANOVA. [file 13073_2014_99_MOESM3_ESM.pdf]
