# Supplementary material for: The conjunctival microbiome in health and trachomatous disease: a case control study
Source: Genome Med. 2014 Nov 15;6(11):99. doi: 10.1186/s13073-014-0099-x (PMC4256740; doi:10.1186/s13073-014-0099-x)

Additional File 4

Comparison of richness and diversity between participants ≤10 and >10 years of age

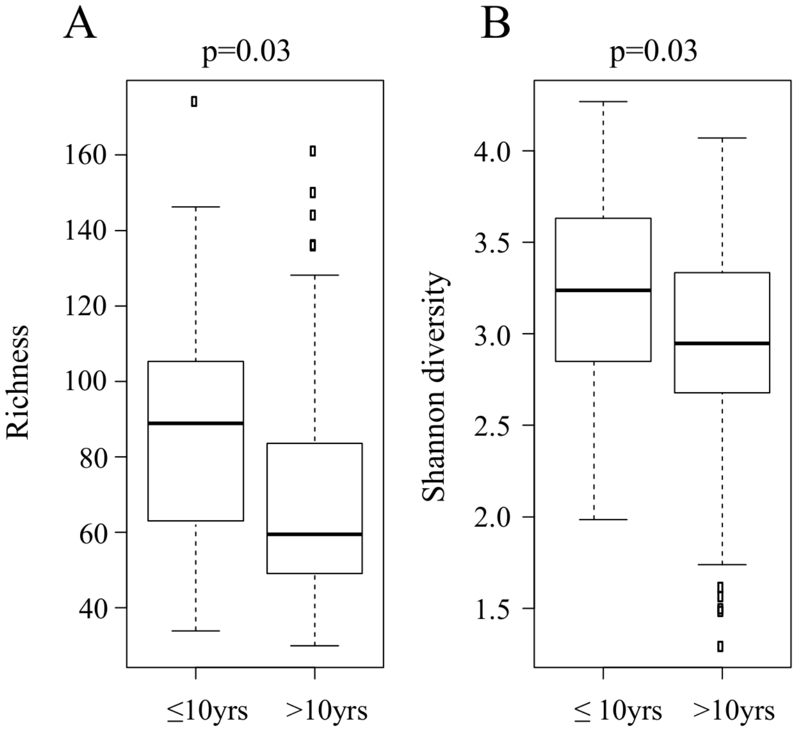

Supplement: Additional file 4: — Effect of age on bacterial community richness and diversity. Boxplots indicate the distribution of (A) richness and (B) Shannon diversity measures in younger (≤10 years) and older (>10 years) participants with normal conjunctivae (F0P0C0). P-values calculated using Wilcoxon rank sum test. [file 13073_2014_99_MOESM4_ESM.pdf]
