# Supplementary material for: The conjunctival microbiome in health and trachomatous disease: a case control study
Source: Genome Med. 2014 Nov 15;6(11):99. doi: 10.1186/s13073-014-0099-x (PMC4256740; doi:10.1186/s13073-014-0099-x)

Additional File 5

Comparison of richness and diversity in participants >10 years of age sampled in the dry versus wet seasons

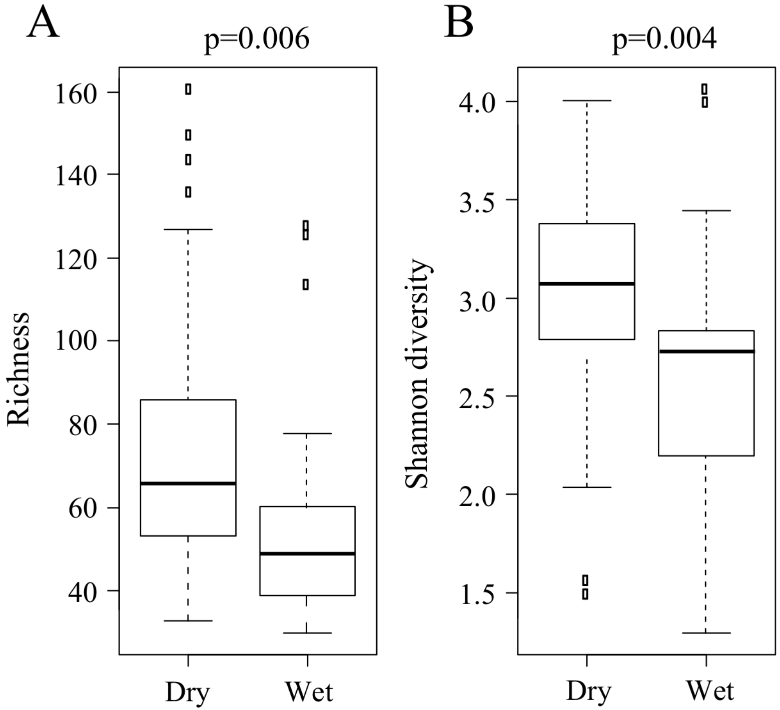

Supplement: Additional file 5: — Effect of season on bacterial community richness and diversity. Boxplots indicate the distribution of (A) richness and (B) Shannon diversity measures in participants >10 years of age with normal conjunctivae (F0P0C0) sampled during the dry and wet seasons. P-values calculated using Wilcoxon rank sum test. [file 13073_2014_99_MOESM5_ESM.pdf]
